# Anti-mitochondrial autoantibodies in systemic lupus erythematosus and their association with disease manifestations

**DOI:** 10.1038/s41598-019-40900-3

**Published:** 2019-03-14

**Authors:** Yann Becker, Renée-Claude Loignon, Anne-Sophie Julien, Geneviève Marcoux, Isabelle Allaeys, Tania Lévesque, Emmanuelle Rollet-Labelle, Hadrien Benk-Fortin, Nathalie Cloutier, Imène Melki, Lihi Eder, Éric Wagner, Martin Pelletier, Hassan El Hajj, Marie-Ève Tremblay, Clémence Belleannée, Marie-Josée Hébert, Mélanie Dieudé, Joyce Rauch, Paul R. Fortin, Eric Boilard

**Affiliations:** 1 Centre de Recherche du CHU de Québec – Université Laval, Département de microbiologie et immunologie, Faculté de Médecine de l′Université Laval, Québec, Qc Canada; 2 Division de Rhumatologie, Département de Médecine, CHU de Québec – Université Laval, Québec, Qc Canada; 3 Département de mathématiques et statistique, Université Laval, Québec, Qc Canada; 4 Women’s College Hospital and University of Toronto, Toronto, ON Canada; 5 Immunology and Histocompatibility Laboratory, Department of Medical Biology CHU de Québec - Université Laval; Department of Microbiology, Infectious Diseases and Immunology, Université Laval, Québec, Qc Canada; 6 Axe maladies infectieuses et inflammatoires, Centre de recherche du CHU de Québec – Université Laval, Québec, Qc Canada; 7 Axe Neurosciences, Centre de Recherche du CHU de Québec – Université Laval, Québec, Qc Canada; 8 Axe Reproduction, Santé de la mère et de l’enfant, Centre de recherche du CHU de Québec – Université Laval, Québec, Qc Canada; 9 Centre de recherche du CHU de Montréal, Montréal Québec, Québec, Qc Canada; 10 Division of Rheumatology, Department of Medicine, Research Institute of the McGill University Health Centre, Montreal, Qc H4A 3J1 Canada

## Abstract

Mitochondria are organelles that govern energy supply and control cell death. Mitochondria also express bacterial features, such as the presence of inner membrane cardiolipin and a circular genome rich in hypomethylated CpG motifs. While mitochondrial extrusion by damaged organs or activated cells is thought to trigger innate immunity, it is unclear whether extracellular mitochondria also stimulate an adaptive immune response. We describe the development of novel assays to detect autoantibodies specific to two distinct components of the mitochondrion: the mitochondrial outer membrane and mitochondrial DNA. Antibodies to these two mitochondrial constituents were increased in both human and murine systemic lupus erythematosus (SLE), compared to controls, and were present at higher levels than in patients with antiphospholipid syndrome or primary biliary cirrhosis. In both bi- and multi-variate regression models, antibodies to mitochondrial DNA, but not whole mitochondria, were associated with increased anti-dsDNA antibodies and lupus nephritis. This study describes new and optimized methods for the assessment of anti-mitochondrial antibodies, and demonstrates their presence in both human and murine SLE. These findings suggest that different mitochondrial components are immunogenic in SLE, and support the concept that extracellular mitochondria may provide an important source of circulating autoantigens in SLE.

## Introduction

The roles of mitochondria in bioenergetics and the control of cell proliferation or death are well-documented^1,2^. Furthermore, mitochondria share several similarities with bacteria^3,4^. Like bacteria, mitochondria are formed of an outer and an inner membrane (inner contains cardiolipin)^4,5^, express formylated peptides^6,7^, and contain a circular genome with hypomethylated DNA CpG motifs, referred to as mitochondrial DNA (mtDNA)^8,9^.

Various cellular lineages are capable of extruding their mitochondria. Activated mast cells^10^, T-cells^11^, eosinophils^12^, hepatocytes^13^, neutrophils^14–16^ and platelets^17,18^, in addition to damaged organs or tissues^7,13,19,20^, release extracellular mitochondria. Mitochondria and their components (*e.g*. N-formylated peptides and mtDNA) are recognized as damage-associated molecular patterns (DAMPs), which activate the innate immune system and elicit an inflammatory response^6,21–23^. Moreover, ATP and reactive oxygen species (ROS), produced by mitochondria are triggers of the nuclear oligomerization domain (NOD)-like receptors and contribute to inflammasome activation^21,22,24^. Extracellular mitochondria have been described in various clinical conditions, including trauma^25,26^, burn injury^27^, cancer^28^, rheumatoid arthritis^17,29^, systemic lupus erythematosus (SLE)^15,16^ and transfusion adverse reactions^17,18,30,31^. Their pro-inflammatory potential is generally thought to occur through activation of Toll-like receptors (TLR), formyl peptide receptors, and cytosolic pathogen recognition receptors, all key components of the innate immune system^6,21–24^.

The adaptive immune system can also recognize mitochondria. This concept is important, as the immune response initiated by mitochondrial DAMPs may be different if the adaptive immune system is also implicated^32,33^. Different sets of anti-mitochondrial antibodies (AMA), namely AMA-M1 to -M9, have been characterized^34^ (recapitulated in Table 1). The AMA-M2, -M4, -M8, and -M9 classes are well-described in primary biliary cirrhosis (PBC)^35–37^, an autoimmune disease characterized by a progressive destruction of the bile ducts due to the infiltration of autoreactive T-cells^38^. These antibodies target distinct mitochondrial proteins notably implicated in oxidative phosphorylation, and their differential induction depends on disease severity or stage. Conversely, AMA-M6 autoantibodies have been described in iatrogenic hepatitis induced by iproniazid^39^, while the AMA-M7 class of antibodies targets mitochondrial epitopes, identified as sarcosine dehydrogenase and enzymes associated with flavin adenine dinucleotide, in patients with cardiomyopathy and myocarditis^40^.

**Table 1:** Various types of anti-mitochondrial antibodies implicated in human diseases.

Type of anti-mitochondrial antibody:	Molecular target(s):	Localization:	Associated disease(s):	Method(s) of detection:	References:
Anti-M1	Cardiolipin	IMM	APS, SLE, secondary syphilis	IIF, ELISA^a^, CFT	^87^
Anti-M2	2-oxoacid dehydrogenase complex	IMM	PBC	IIF, ELISA^a^, CFT	^58^
Anti-M3	*Unknown*	OMM	Venocuran-induced PLE	IIF, CFT	^55^
Anti-M4	Sulfite oxidase	OMM	PBC	ELISA^b^, CFT	^88^
Anti-M5	*Unknown*	OMM, IMM	APS, SLE, SS, haemolytic anemia	IIF, CFT	^56^
Anti-M6	Monoamine oxydase B	OMM	Iproniazid-induced hepatitis	IIF, ELISA^b^, CFT	^39, 89^
Anti-M7	Sarcosine dehydrogenase	IMM	Cardiomyopathies	ELISA^a–c^	^90^
Anti-M8	*Unknown*	OMM	PBC	CFT	^89^
Anti-M9	Glycogen phosphorylase	OMM	PBC	ELISA	^91, 92^

^a^ELISA performed on sub-mitochondrial particles (sonicated “crude” mitochondria).

^b^ELISA performed on purified antigen.

^c^The exact antigen recognized by M7 antibodies is yet to fully characterize.

APS, anti-phospholipid syndrome; CFT, complement fixation test; ELISA, enzyme-linked immunosorbent assay; IIF, indirect immunofluorescence on rodent and/or human tissues; IMM, inner mitochondrial membrane; OMM, outer mitochondrial membrane; PBC, primary biliary cirrhosis; PLE, pseudolupus erythematosus; SLE, systemic lupus erythematosus; SS: Sjögren syndrome.

SLE is an autoimmune disease characterized by the presence of circulating antigen-autoantibody immune complexes and inflammation in multiple organs and tissues. In SLE, neutrophils were shown to release mtDNA through the generation of ROS, a process leading to activation of the DNA sensor stimulator of interferon genes (STING) and type-I IFN production^15,16^. Studies showed that anti-mtDNA antibodies (AmtDNA) are induced in a subset of SLE patients, suggesting that extruded mtDNA could be a relevant source of antigen for the anti-double stranded DNA (anti-dsDNA) antibodies that prevail in SLE^16,41,42^. Clinically, anti-DNA antibodies are screened initially by indirect immunofluorescence using human epithelial type 2 cells (HEp-2) and enzyme immunoassay using double-stranded DNA^43^. Presence of anti-DNA antibodies can also be assessed by indirect immunofluorescence using *Crithidia luciliae*, an hemoflagellate parasite of blow-flies that possesses a uniquely large mitochondrion that contains a high concentration of DNA, the kinetoplast^44–46^. Another method commonly used is Farr radioimmunoassay, which involves the precipitation of antibody-bound radiolabeled DNA and its detection with a scintillation counter^47^.

Antibodies targeting mitochondrial components other than mtDNA are also found in SLE. They include anti-cardiolipin [AMA-M1, also known as anti-cardiolipin antibodies (ACA)], anti-60kDa heat shock protein (anti-HSP60), as well as AMA-M3 and AMA-M5 antibodies. AMA-M1 antibodies recognize cardiolipin and were originally identified in syphilis-infected patients. Anti-cardiolipin antibodies are also found in patients with SLE and antiphospholipid syndrome (APS), resulting in false positive results in earlier syphilis detection assays^34,48^.

Interestingly, cardiolipin is a phospholipid that is found uniquely in the inner mitochondrial membrane in eukaryotic cells. However, damaged mitochondria may externalize their cardiolipin^49^, that may become accessible and induce the development of antibodies to cardiolipin. Clinically, the presence of ACA is associated with a greater risk of thrombotic events and thrombocytopenia^50,51^.

Another mitochondrial antigen, HSP60, is a mitochondrial chaperonin implicated in mitochondrial protein import^52^. Patients with SLE have antibodies against HSP60^53^, and their presence (when concomitant with anti-phospholid antibodies) is associated with vascular events^54^.

AMA-M3 were described in patients using a drug called venocuran who developed a drug-induced syndrome (“pseudolupus”) with clinical manifestations of arthralgia, fever, serositis, and lymphopenia. The immunological profile of pseudolupus is characterized by the absence of antinuclear antibody (ANA), but the presence of AMA-M3^55^. The antigenic target of AMA-M3 is different than that of the other AMA classes described in PBC^55^. It is resistant to trypsin and it is extracted by solvent, pointing to its lipid nature. AMA-M3 are no longer encountered clinically since the withdrawal of the drug venocuran.

AMA-M5 comprise another class of anti-mitochondrial antibodies identified in patients with SLE, as well in APS, Sjögren syndrome, recurrent fetal loss, and hemolytic anemia^56^. The precise antigenic target(s) of AMA-M5 is undefined, but lack of competition by cardiolipin-containing liposomes suggests that it is distinct from the target of AMA-M1^57^. Indirect immunofluorescence on human or rodent tissues is used to identify anti-M5 antibodies^58^. While immunofluorescence and complement fixation test revealed that only 2% of SLE patients were positive for AMA-M5, approximately 40% were positive by enzyme-linked immunosorbent assay (ELISA)^59^. However, in the latter approach, mitochondria were only partially purified and were sonicated, thus revealing antigenic epitopes that might not be recognized in tissues^60^.

Emerging evidence supports the liberation of mitochondria by activated cells and their potential contribution to inflammation in SLE^15,16^. Identification of mitochondrial antigens recognized by autoantibodies in SLE may provide information on the roles of extracellular mitochondria in autoimmunity and systemic inflammation. Herein, we developed new methods to detect the presence of two distinct types of circulating AMA: anti-whole mitochondria antibodies (AwMA) and AmtDNA. We then determined their usefulness in a murine model of SLE, as well as in a cohort of SLE patients. We evaluated these AMA in parallel with antibodies to the mitochondrial chaperonin HSP60 (a known mitochondrial target in SLE), and determined the associations of these autoantibodies with disease characteristics. To our knowledge, this is the first study that examines the association of these AMA with disease manifestations in SLE.

## Results

**Figure 1:**
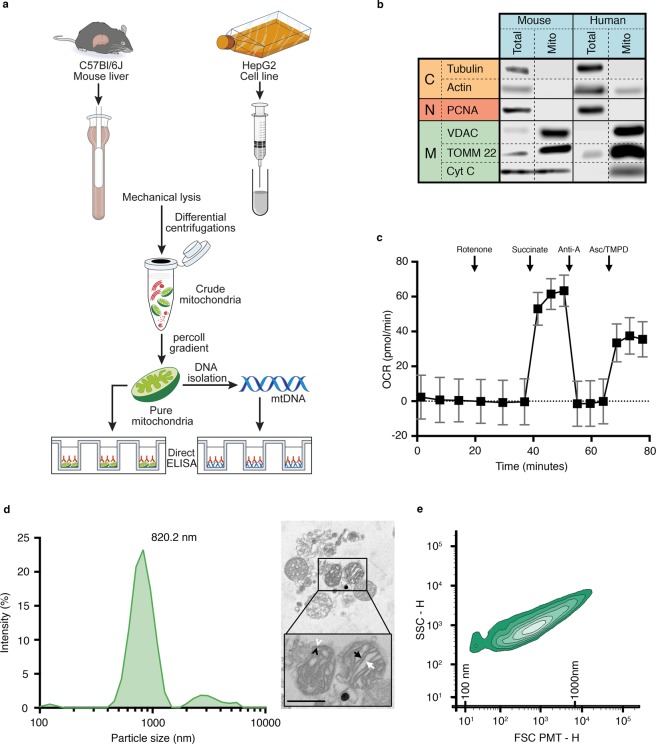
Assessment of the mitochondrial preparations. (**a**) Mitochondria were isolated from either mouse liver or human Hep-G2 cell line by differential centrifugations and further purified by ultracentrifugation against Percoll gradient. Alkaline lysis was performed to retrieve mtDNA. Pure mitochondria or mtDNA were used as coating antigens in direct ELISAs. (**b**) Cytoplasmic (C), nuclear (N) and mitochondrial (M) markers were assessed by western blotting in murine (left) and human (right) mitochondrial preparations (25 µg protein per lane). Results are representative of three distinct preparations. Blots separated by dashed lines are non-contiguous but from same membrane. Blots separated by full lines were performed on distinct membranes; (**c**) Functionality of murine mitochondria was determined by measurement of the oxygen consumption rate (OCR) of 10 µg mitochondria treated successively with 2 µM rotenone, 10 mM succinate, 40 µM antimycin A and 100 µM N,N,N’,N’-Tetramethyl-p-phenylenediamine (TMPD) along with 10 mM ascorbate (Asc). (**d**) The hydrodynamic size of the murine mitochondria was determined using zetasizer nano ZS (n = 3, left panel) and their morphology was visualized by electron microscopy (right panel). Inner membrane (black arrowhead), outer membrane (white arrowhead), cristae (black arrow), mitochondrial matrix (white arrow) are presented (scale bar account for 500 nm); (**e**) Size representation of purified murine mitochondria using high sensitivity-flow cytometry. Double-positive mitochondria (Mitotracker^+^ TOMM22^+^) were used for quantification. Silica beads were used to determine 100–1000 nm size scale. Data are mean ± SD. Anti-A: antimycin A; CytC: cytochtome C; FSC: forward scatter; Mito: mitochondria PCNA: proliferating cell nuclear antigen; SSC: side scatter; Total: total starting material; TOMM22: translocase of the outer mitochondrial membrane; VDAC: voltage-dependent anion channel.

Different methodologies exist for the isolation of mitochondria, and several improvements have been introduced in recent years to enhance their purity and quality. We used a combination of previously published protocols to obtain highly purified mitochondria^61,62^. Mitochondria were isolated from mouse liver or a human hepatocyte cell line (Hep-G2) using a combination of previously published protocols^61,62^ (Fig. 1a). The Percoll gradient included in the purification protocol eliminated contaminants from the endoplasmic reticulum and the proteasome (Supplementary Fig. 1a)^62^. The purity of the mitochondrial preparations was high, based on the extremely low content of cytosolic and nuclear proteins, and the enrichment of three mitochondrial proteins [voltage-dependent anion channel (VDAC), cytochrome C, and translocase of the outer membrane 22 (TOMM22)] (Fig. 1b). The isolated mitochondria maintained their cytochrome C (Fig. 1b) and respiratory functions, suggesting that integrity was conserved during the isolation process (Fig. 1c).

Mitochondria were homogeneous in size, with a main peak detected at 820 nm by dynamic light scattering (Fig. 1d, left panel), and retained their canonical morphology when observed in electron microscopy (910 ± 210 nm) (Fig. 1d, right panel). High sensitivity flow cytometry, which permits the identification of submicron particles, was used as a quantitative approach. We estimated that 6.6 ± 1.9 × 10^6^ mitochondria (Mitotracker^+^ TOMM22^+^) (or 4.33 ± 1.17 µg mitochondrial proteins) could be isolated per mg of mouse liver (n = 6), which was sufficient to prepare approximately 400 wells in half-area 96-well microplates using one mouse liver (Fig. 1e). The yield obtained with Hep-G2 cells was lower, as 3.5 ± 0.18 µg of mitochondrial protein were isolated per 10^6^ Hep-G2 cells (7.0 × ± 2.1 × 10^8^ Hep-G2 cells were harvested for each 175 cm^2^ flask at confluence), which can be used to prepare approximately two half-area 96-well microplates.

**Figure 2:**
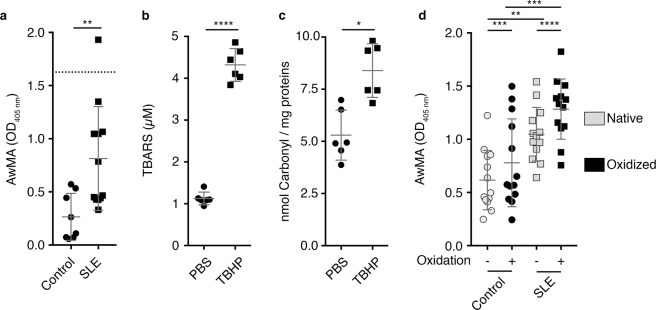
Antibodies targeting mitochondrial antigens are produced in a murine model of SLE. (**a**) Elevated levels of anti-whole mitochondria antibodies (AwMA) were detected by direct ELISA in sera (1:150) from an inducible murine model of systemic lupus erythematosus (SLE) compared to control mice. An isotype-matched monoclonal mouse anti-translocase of the outer mitochondrial membrane 22 (TOMM22) antibody (clone IC9-2. 4 µg/mL) was included as a positive assay control (dotted line). (Control: N = 8, SLE: N = 12, Student’s t-test); (**b**) Lipid peroxidation following *in-vitro* oxidation of the mitochondria by 500 µM tert-buthyl hydroperoxide (TBHP) was quantified by thiobarbituric reactive substances (TBARS) assay (N = 3, Wilcoxon test); (**c**) Protein oxidation was determined by carbonyl assay (n = 6, Wilcoxon test); (**d**) The effect of oxidation of mitochondrial epitopes on their recognition by serum AwMA (1:20) was assessed by direct ELISA, using either native (grey symbols) or oxidized mitochondria (black symbols) as coating antigens (N = 13, two-way ANOVA with multiple comparisons; Sidak’s correction). All experiment presented in the figure were performed using mouse mitochondria. Data are mean ± SD. *p < 0.05. **p < 0.01. ***p < 0.001. ****p < 0.0001.

The quantity of purified mitochondrial protein (12.5 µg mitochondrial protein/well) required for coating half-area 96-well microplates ELISA plates was optimized using increasing concentrations of mitochondria (Supplementary Fig. 1b). Wells were saturated with phosphate buffered saline (PBS) containing fetal calf serum (FCS) and gelatin, which proved optimal for blocking nonspecific binding of antibodies in comparison to non-fat dry milk or bovine serum albumin (data not shown). We used an inducible murine model of SLE to determine whether AwMA could be detected in serum^63^. This model is known to produce autoantibodies to nuclear and cellular antigens^63^, but the presence of AwMA has never been explored. We found high serum levels of AwMA in this induced murine lupus model, compared to healthy control mice (Fig. 2a).

Reactive oxygen species are generated under inflammatory conditions, and were reported during the release of mitochondria^15,16^. Thus, we assessed whether oxidation of mitochondria could impact mitochondrial recognition by AwMA. Isolated mitochondria were treated with increasing concentrations of the oxidant tert-butyl hydroperoxide (TBHP), and the oxidized protein and lipid contents were confirmed using commercial assays (Fig. 2b,c and Supplementary Fig. 2). We found that oxidation had no or very little impact on recognition of mitochondria by SLE antibodies (Fig. 2d) (Fold increase: 1.2 ± 0.2). The data suggest that mitochondria are immunogenic in SLE regardless of the oxidation status of their antigens.

We next used our quantitative AwMA ELISA to screen human sera. We included 175 SLE patients and 43 healthy controls (76% female, mean age 42 ± 12) (Table 2). We also evaluated sera from APS patients (n = 12), given the high levels of anti-cardiolipin antibodies (AMA-M1) in APS, as well as sera from PBC patients (n = 12) confirmed positive for AMA by indirect immunofluorescence on mouse stomach/kidney slides (MSK).

**Table 2:** Demographics and clinical characteristics (ACR criteria) for SLE patients included in the study (n = 175).

Characteristics	SLE patients
Age	
Range, years	20–78
Mean ± S.D, years	47 ± 15
Disease duration	
Range, years	0–57
Mean ± S.D, years	18 ± 12
Gender, female, n (%)	175 (100)
Thrombotic events, n (%)	35 (20)
SLEDAI-2K ≥ 4, n (%)	57 (33)
SDI ≥ 1, n (%)	124 (71)
Increased anti-dsDNA, n (%)	59 (34)
Lupus nephritis, n (%) (n = 172)	67 (39)
Currently Prescribed Medication, n (%)	
Anticoagulation or anti-platelet (n = 174)	40 (23)
Antimalarial	127 (73)
Prednisone	81 (46)
Lipid lowering	26 (15)
Diabetes medication	6 (3)
Malar rash	127 (72.6)
Discoid rash	24 (13.7)
Photosensitivity	113 (64.6)
Oral ulcers	108 (61.7)
Arthritis (≥2 peripheral joints)	151 (86.3)
Serositis	67 (38.3)
Neurologic disorder (seizure or psychosis)	24 (12.0)
Renal disorder^A^	100 (57.1)
eGFR (n = 160)	
Range, mL/min/1.73 m^2^	17–121
Mean ± S.D, mL/min/1.73 m^2^	84.38 ± 24.70
<60 mL/min/1.73 m^2^, n (%)	26 (16.3)
Hematologic disorder^B^	155 (88.6)
Immunologic disorder^C^	159 (90.9)
Anti-nuclear antibodies (ANA)	170 (97.1)
American College of Rheumatology criteria (ACR) score	
Range	03-Nov
Mean ± S.D	6.83 ± 1.62

^A^>0.5 g per day of protein in urine or cellular cast or end-stage renal disease.

^B^Hemolytic anemia (low red blood cell count) or leukopenia (White blood cells < 4000/µl), lymphopenia (<100 000/µl) in the absence of offending drug.

^C^Positive anti-Smith, anti-dsDNA, antiphospholipid antibody and/or false positive serological test for syphilis.

eGFR: estimated glomerular filtration test.

**Figure 3:**
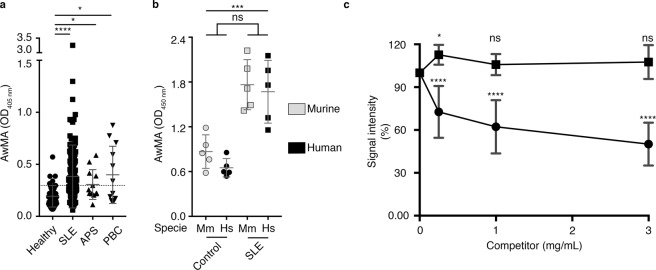
Detection of anti-mitochondrial antibodies in SLE patients and specificity of the assay. (**a**) Increased amounts of anti-whole mitochondria antibodies (AwMA) were detected by direct ELISA in sera (1:150) from systemic lupus erythematosus (SLE), anti-phospholipid syndrome (APS) and primary biliary cirrhosis (PBC) patients. Healthy: N = 43. SLE: N = 175. APS: N = 12, PBC: N = 12. The dotted line corresponds to the cutoff value as determined by Youden’s Index (see Table 5). Kruskal-Wallis test with multiple comparisons to healthy donors; Dunn’s correction. (**b**) No significant differences were detected by direct ELISA when either murine (Mm, gray symbols) or human mitochondria (Hs, black symbols) were used as coating antigens to detect AwMAs in control and SLE patient sera (1:100). Two-way ANOVA. (**c**) AwMA binding to coating mitochondria is inhibited in presence of mitochondria (filled circles) but not by red blood cells microparticles (filled squares). Two-way repeated measures ANOVA with multiple comparison (Dunnett’s correction) to signals detected without competitors (i.e. 100%). Experiments presented in panels 3 a and 3 c were performed using mouse mitochondria, the experiment presented in panel 3 b was performed in parallel on murine and human mitochondria. Data are mean ± SD. Not significant (ns): p > 0.05. *p < 0.05. ***p < 0.001. ****p < 0.0001. Hs: *Homo sapiens*; Mm: *Mus musculus*.

Given the higher yield and purity of the mitochondria isolated from mouse liver, and the fact that mice are readily accessible in most research laboratories, we used intact murine mitochondria as coating antigens in our assay for the detection of autoantibodies targeting the outer mitochondrial membrane in humans. We found that AwMA were present in all healthy controls, but at much lower levels than those encountered in a large proportion of the SLE patients. SLE patients were more frequently positive and at higher levels for AwMA than healthy controls. APS and PBC patients also presented a significant increase in AwMA compared to healthy donors but signals detected for these patients were lower than those measured in SLE patients (Fig. 3a). As the ELISA is performed on intact mitochondria, these results suggest that AwMA are induced in SLE, and recognize autoantigens on the outer mitochondrial membrane that are distinct from the epitopes in APS (cardiolipin) and PBC (pyruvate dehydrogenase complex E2-component, PDC-E2), both located in the mitochondrial inner membrane. Consistent with this, we identified PBC patients positive for AMA when submitochondrial particles (*i.e*. sonicated mitochondria) were used as coating antigens, suggesting that certain antigens relevant to PBC may be exposed in these conditions (Supplementary Fig. 3). Human and murine mitochondria were also compared in our assay using sera from a subset of SLE patients. Both sources of mitochondria (human Hep-G2 cells and mouse liver) were similarly recognized by human AwMA (Fig. 3b), suggesting that antigenic epitopes may be conserved across these species. Hence, mitochondria from murine, bovine and porcine tissues are routinely used to assess AwMA in humans, suggesting that interspecies differences play a negligible role, if any, in mitochondrial antigenicity.

Membrane-bound vesicles in the extracellular milieu, known as extracellular vesicles or microparticles, are proposed contributors to the antigenic load in SLE^64–66^. To determine whether AwMA could also recognize microparticles derived from membranes from cells or particles other than mitochondria, we utilized red blood cell microparticles (_RBC_MP) as blood-borne microparticles devoid of mitochondria as a competitor in our AwMA-ELISA, and compared it to extracellular mitochondria in solution. Whereas increased concentrations of competing mitochondria decreased AwMA binding by up to 49.84 ± 15.01%, _RBC_MP showed no inhibition of binding of the SLE antibodies in our assay (Fig. 3c). While these results suggest that the antibodies detected in the AwMA-ELISA might have a preferred substrate originating in mitochondrial membrane, we cannot exclude the possibility that other membrane bound microparticles or even cells may also be recognized by AwMA, given the probable occurrence of numerous protein and non-protein antigens in mitochondria.

**Figure 4:**
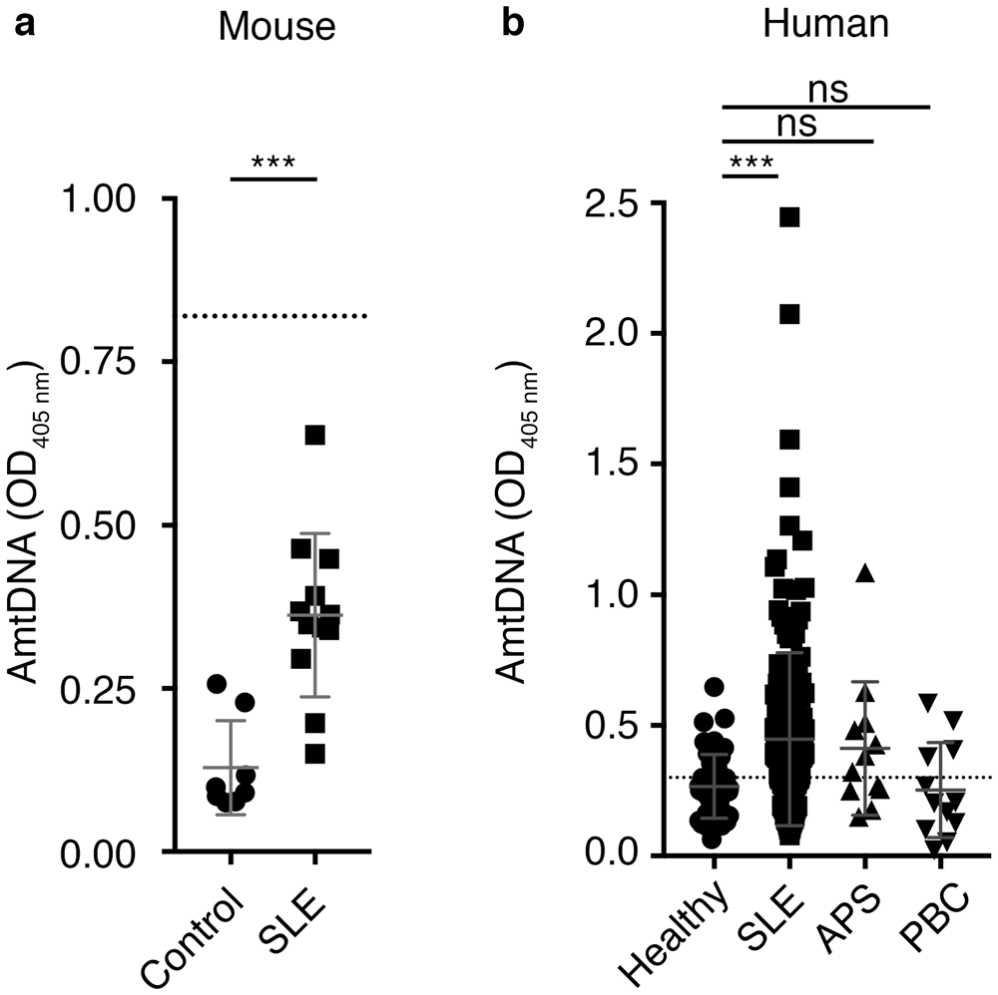
Antibodies targeting mitochondrial DNA in SLE. (**a**) Anti-mitochondrial DNA antibodies (AmtDNA) are measured by direct ELISA in sera (1:50) from a mouse model of systemic lupus erythematosus (SLE) and control mice (Control: N = 8, SLE: N = 12, Student’s t-test). An isotype-matched monoclonal mouse anti-DNA antibody (clone 35I9 DNA, 10 µg/mL) was included as a positive assay control (dotted line). (**b**) Elevated levels of AmtDNA are observed in sera (1:150) from SLE but not from anti-phospholipid syndrome (APS) or primary biliary cirrhosis (PBC) patients. Healthy: N = 43. SLE: N = 175. APS: N = 12. PBC: N = 12. The dotted line corresponds to the cutoff value as determined by Youden’s index (see Table 5). Kruskal-Wallis test with multiple comparisons to controls/healthy donors; Dunn’s correction). All experiment presented in the figure were performed using mouse mtDNA. Data are mean ± SD. Not significant (ns): p > 0.05. ***p < 0.001. PBC: primary biliary cirrhosis.

To determine whether mtDNA also represents an antigenic target of SLE autoantibodies, we isolated mtDNA from crude mitochondria preparations, using standard DNA extraction by alkaline lysis. Digestion of the mtDNA by *Hae II*, a restriction enzyme with a single restriction site on the murine mitochondrial genome (Supplementary Fig. 4a) yielded a single fragment of 16,569 base pairs, indicating the isolation of mtDNA with its circular conformation. As expected, digestion by *Pst I* generated two fragments of 12,751 and 3818 base pairs, further confirming the expected size of the isolated mtDNA. Moreover, we confirmed enrichment of mtDNA relative to genomic DNA (Supplementary Fig. 4b). Up to 1.55 ± 0.35 µg mtDNA was obtained for each mg of mitochondrial protein used. Plate adhesion of different concentrations of mtDNA was enhanced by using plates pre-treated with protamine sulfate, and binding specificity was increased by blocking the plates with a PBS solution containing FCS and gelatin (Supplementary Fig. 4c). Of interest, sera from mice with induced SLE were positive for AmtDNA, compared to control mice (Fig. 4a). Moreover, AmtDNA was significantly increased in SLE patients, but not in patients with APS or PBC, relative to healthy controls (Fig. 4b).

Little is known about the association of AwMA and AmtDNA with the clinical characteristics of SLE. We assessed whether AwMA and AmtDNA were associated with disease manifestations in 175 SLE patients for whom detailed clinical information was available. AwMA levels correlated with AmtDNA levels in SLE patients (r_s_ = 0.23, p = 0.003), but not in healthy controls (r_s_ = 0.15, p = 0.33). In contrast, AwMA did not correlate with antibodies against other mitochondrial antigens (*i.e*. HSP60 and cardiolipin) and was not found to be associated with clinical outcomes (Tables 3 and 4). Interestingly, AmtDNA was associated with both increased anti-dsDNA antibodies (p = 0.02) and with a history of lupus nephritis (p = 0.007), but not with any of the other clinical outcomes (Table 4). When the duration of the disease, the age of the patients, their BMI, the use of prednisone and/or antimalarial drugs, and circulating cholesterol LDL were taken into account, the associations of AmtDNA with increased anti-dsDNA antibodies and lupus nephritis remained significant in a multivariate logistic regression (p = 0.01 for both), indicating an association between AmtDNA, and these two clinical parameters. However, AmtDNA did not correlate with anti-dsDNA as measured by Farr assay in the cohort, suggesting that the results measured by our AmtDNA-ELISA and those obtained by Farr assay may not be redundant. Cut-off values were identified for AwMA and AmtDNA (Figs 3, 4 and Table 5). Our two ELISAs displayed high specificities (AwMA: 0.88; AmtDNA: 0.74) and allowed us to efficiently discriminate SLE patients from healthy donors (p < 0.001 for both AmtDNA and AwMA). Using these values, we determined that 110 patients were positive for AmtDNA (62.86%) and 101 patients (57.71%) were positive for AwMA.

**Table 3:** Intercorrelations of anti-mtDNA, anti-whole mitochondria, anti-dsDNA and anti-cardiolipin antibodies in SLE patients (n = 175).

	AmtDNA^A^	AwMA^A^	DNA (Farr)^A^	ACA (+/−)^B^
Anti-HSP60	0.07 p = 0.37	0.10 p = 0.21	0.02 p = 0.81	(+) 0.28 ± 0.52 (−) 0.52 ± 0.53 p = 0.08
AmtDNA	—	0.23, p = 0.003	0.05 p = 0.46	(+) 0.33 ± 0.17 (−) 0.37 ± 0.27 p = 0.40
AwMA	—	—	0.10 p = 0.19	(+) 0.32 ± 0.20 (−) 0.33 ± 0.23 p = 0.51

^A^Values are presented as Spearman correlation coefficient and p-value.

^B^Values presented as median ± IQR and Wilcoxon test p-value for patient positives (+) or negatives (−) for ACA.

ACA: anti-cardiolipin antibodies; AwMA: anti-whole mitochondria antibodies. AmtDNA: anti-mitochondrial DNA antibodies; DNA Farr: quantification of anti-dsDNA antibodies by Farr assay; HSP60: heat-shock protein 60 KDa.

**Table 4:** AmtDNA and AwMA associations with clinical manifestations in SLE patients (n = 175).

Clinical Outcomes	AmtDNA		AwMA	
	OR (CI)	p	OR (CI)	p
Thrombotic events	0.43 (0.10–1.79)	0.25	0.27 (0.04–1.99)	0.2
	[0.35 (0.07–1.67)]*	[0.19]	[0.21 (0.02–1.82)]	[0.15]
SLEDAI-2K ≥ 4	0.96 (0.37–2.51)	0.93	1.34 (0.49–3.69)	0.57
	[1.01 (0.33–3.05)]	[0.99]	[1.08 (0.37–3.14)]	[0.89]
SDI ≥ 1	0.91 (0.35–2.42)	0.86	0.85 (0.30–2.40)	0.76
	[0.93 (0.32–2.68)]	[0.90]	[0.69 (0.23–2.09)]	[0.52]
Increased anti-dsDNAA	3.34 (1.22–9.16)	0.02	1.15 (0.42–3.16)	0.79
	[3.94 (1.33–11.69)]	[0.01]	[1.16 (0.40–3.35)]	[0.79]
Lupus nephritis	4.45 (1.50–13.20)	0.007	1.06 (0.39–2.90)	0.91
	[4.60 (1.41–14.99)]	[0.01]	[0.97 (0.34–2.73)]	[0.95]

^A^Occurrences of patients with anti-dsDNA antibodies above the clinical threshold.

AwMA: anti-whole mitochondria antibodies. AmtDNA: anti-mitochondrial DNA antibodies. SDI: lupus severity disease index. SLEDAI-2K: systemic lupus erythematosus disease activity index - 2000. OR (CI): Odds ratios (95% Wald Confidence Interval). P from logistic regressions.

*Bivariate results (N = 175) are followed by multivariate results in square brackets (N = 169).

**Table 5:** Performance of cut-off values for AwMA and AmtDNA (Healthy donors: n = 43, SLE: n = 175).

	Cutpoint (OD_405 nm_)	Sensitivity	Specificity	PPV	NPV	AUC
AwMA	0.30 (0.17–0.32)	0.58 (0.50–0.65)	0.88 (0.75–0.96)	0.95 (0.89–0.98)	0.34 (0.25–0.43)	0.80 (0.73;0.87)
AmtDNA	0.30 (0.25–0.45)	0.63 (0.55–0.70)	0.74 (0.59–0.87)	0.91 (0.84–0.95)	0.33 (0.24–0.43)	0.71 (0.63;0.79)

AwMA: anti-whole mitochondria antibodies. AmtDNA: anti-mitochondrial DNA antibodies. AUC: area under the curve. OD: optical density. PPV: Positive Predictive Value. NPV: Negative Predictive Value. NPP: Negative Predictive Value.

Thus, although more work is needed to explore the clinical associations of AmtDNA and AwMA in a larger lupus population and over time, different subsets of mitochondrial epitopes (*i.e*. mtDNA *vs*. outer membrane antigens) appear to measure different immune responses in SLE patients and may be associated with distinct disease characteristics.

## Discussion

Mitochondrial DAMPs are known to stimulate the innate immune response, but it is less clear whether mitochondrial antigens stimulate an antibody response and whether these antibodies impact the inflammatory reaction. Interestingly, in PBC, a paradigm of true organ-specific autoimmunity, evidence points to involvement of both innate and adaptive immunity with a specific antibody response to mitochondrial antigens^67^.

Although the prevalence of AMA in SLE was reported several decades ago^59^, this observation was not pursued. Our study confirms the presence of AwMA (directed against the mitochondrial surface and not redundant with that of PBC) and AmtDNA (directed against mtDNA) in patients with SLE. However, it remains to be established whether AwMA are pathogenic initiators of the auto-immune process or whether they are consequences of an a priori cell activation or injury with subsequent release of mitochondria in the extracellular milieu. These “free mitochondria” could subsequently become antigenic in predisposed individuals and be a marker of cell or tissue injury. They could also be a cause of further immune activation through the formation of circulating or *in-situ* immune complexes, constituting a secondary trigger of inflammation. The recognition of mitochondria by antibodies could also implicate Fc receptors and thus modulate a distinct immune response. For example, the intravenous injection of mtDNA into mice failed to induce proteinuria and kidney damage^68^, but the response may differ in a recipient with circulating antibodies to mitochondria.

Epitope modification, such as oxidation, can impact its antigenicity^69^. Notably, there are reports suggesting that the oxidation of mtDNA occurs during its extrusion from cells, and that the oxidized form is pathogenic^15,16^. Our findings using *in vitro* oxidation of the mitochondria suggest that mitochondrial epitopes, regardless of oxidation status, are targeted by autoantibodies in SLE. However, this does not exclude the possibility that oxidized mitochondria may be more antigenic in other pathogenesis, such as APS, or more efficient at promoting responses if recognized by the innate immune system. Moreover, it is not excluded that following oxidation, mitochondrial swelling and potential formation of pores within the mitochondrial membrane might have revealed new antigens detected in our anti-whole mitochondria ELISA using TBHP-treated mitochondria, but this treatment may also have caused a release of existing antigens that could have been lost in this modified assay, thus explaining the apparent absence of impact of oxidation of the antigenicity of the mitochondria^70^. Future investigations will be needed to determine the relative targeting of native versus oxidized forms of organelle components by immune cells. We could not verify the role of oxidation on the recognition of mtDNA by autoantibodies due to spontaneous oxidation of isolated mtDNA, an observation also made by other groups despite extreme preventive measures taken to maintain the molecules under their reduced form^71^. Thus, it is likely that AmtDNA and anti-dsDNA antibodies routinely measured in clinical testing both evaluate antibodies to oxidized DNA.

We observed that AwMA levels were more elevated in SLE than in PBC. PBC patients, however, were positive for AMA when sonicated mitochondria were utilized in our ELISA. These observations are consistent with the fact that pyruvate dehydrogenase complex E2 (PDC-E2), the immunodominant epitope in PBC, is located in the mitochondrial inner membrane. Our AwMA-ELISA measures antibodies to epitopes on intact mitochondria, in which inner membrane epitopes remain unavailable^72^. This contrasts with the sonicated mitochondria used in previous studies on AMA in SLE^59^, which contained inverted membranous structures and thus revealed antigens located in the mitochondrial inner membrane^60^. Furthermore, we found that AwMA levels correlated with the levels of AmtDNA, but not with other antibodies directed at antigens located within the inner membrane of the organelle (e.g. cardiolipin or HSP60), pointing to the existence of mitochondrial antigens that remain to be identified^73^. Our study provides simple and quantitative assays for assessment of two types of AMA (i.e. AwMA and AmtDNA) likely specific to SLE. Further research is required to identify the mitochondrial antigen(s) and epitope(s) of our AMA assay in SLE.

Our analyses of AmtDNA antibodies in human sera showed an association with anti-dsDNA as well as with lupus nephritis, consistent with the documented associations between anti-dsDNA antibodies detected by Farr assay and lupus nephritis^74^. While dsDNA used in Farr assays is usually isolated from plasmids (*i.e. E. coli*) and thus may share similarities with mtDNA (*e.g*. hypomethylated CpG motives, circular tertiary structure)^75^, the Farr assay has been described for its specificity in the detection of high avidity antibodies of all classes (IgG and IgM, for instance). Our data suggest that the Farr assay probably detects AmtDNA. Consistent with this, nuclear DNA (nDNA) efficiently competed in our AmtDNA ELISA assay (Supplementary Fig. 5a), further pointing to a certain degree of redundancy between anti-dsDNA and AmtDNA. When nuclear and mtDNA were treated with S1 nuclease, which digests potential single-stranded regions, both could compete in our AmtDNA ELISA assay (Supplementary Fig. 5b), which suggests that most of the antigenicity of mtDNA is provided by its double-stranded conformation. There might exist subsets of antibodies that preferentially recognize mtDNA versus genomic DNA, but there is no evidence of their occurrence at this stage. As extracellular mtDNA is reportedly associated with various pathologies, including trauma^25,26^, burn injury^27^, cancer^28^ and rheumatoid arthritis^17,29^, it is tempting to postulate that extruded mtDNA, beyond its role as a pro-inflammatory signal for the innate immune system, represents a significant antigenic load available for the formation of immune complexes.

In this study, we focused our attention on immunoglobulin (Ig) G (IgG), but the assays can be modified easily to quantify specific IgG subclasses (e.g. IgG1, IgG2a, IgG2b) or other isotypes (e.g. IgA and IgM), which may reveal distinct functions of these antibodies in disease. Both AwMA and AmtDNA were detected at low levels in healthy individuals, suggesting that these antibodies are further induced during SLE pathogenesis. These antibodies might be part of a pool of naturally occurring autoantibodies that are thought to contribute to the continual clearance of membrane vesicles in the circulation^76,77^. Protective natural autoantibodies have been described predominantly as IgM, but other isotypes such as IgG or IgA have also been reported^78–80^. It is thus tempting to postulate that antibodies targeting mitochondrial epitopes are present in healthy individuals and might be involved in clearance of extracellular mitochondria. A profound change in the balance of natural IgM and pathogenic IgG against mitochondrial epitopes may in part explain the pathogenesis in SLE.

While these observations require confirmation in an independent cohort of patients, they suggest that, in SLE, the adaptive immune system recognizes mitochondrial organelles. Furthermore, our findings suggest that clinical associations may differ according to antibody recognition of inner (*e.g*., ACA) versus outer mitochondrial (*e.g*., AwMA) membrane components. This leads to the postulation that pathogenicity of these antibodies may depend on whether extracellular mitochondria are intact or not. Evaluation of antibodies to mitochondrial components in SLE may provide novel information on patients, such as their risk for developing nephritis. If these findings are confirmed in a large prospective cohort of SLE patients, AwMA and AmtDNA may prove useful in predicting disease activity and disease severity, and in stratifying SLE patients. The quantification of mitochondrial antibodies may thus open the way to novel directions in autoimmune disease research and may be useful for achieving a better understanding of disease mechanisms.

## Material and Methods

### Study approval

#### Patient population

The human sera tested in this study were obtained from a University Health Network research ethics board (REB) approved study of SLE and APS patients in Toronto as well as from healthy controls and PBC patients recruited from CHU de Québec – Université Laval REB approved studies in Quebec City. SLE patients had to meet the 1982 ACR classification criteria for SLE revised in 1997^81,82^ and APS patients met the 1999 Sapporo criteria for APS revised in 2006^83,84^. For SLE, consecutive female patients from the University of Toronto Lupus Clinic (UTLC) were approached and provided consent between August 2010 and October 2011 to be part of a study on the role of fatty acids and cardiovascular disease in lupus. They provided one blood specimen and had their anonymized clinical data linked to their biospecimen. Similarly, APS patients seen at the rheumatology clinic in Toronto were approached following a similar procedure. All remaining biospecimen could be used for future studies on biomarkers of lupus as per the original subjects’ consent. Healthy control volunteers were recruited as part of study on markers of inflammation if they had no known illnesses and did not have infectious symptoms at the time of the blood draw. Age and sex were collected at that time. PBC patients met the 2009 PBC classification criteria, revised in 2018^38,85^ including the positivity for anti-mitochondrial antibodies.

### Data from clinical laboratories

For SLE patients, anti-dsDNA were measured using the Farr assay (laboratory cut-off of 30%) and the anti-cardiolipin (IgG and IgM – laboratory cut-offs of 40 GPL or MPL units) were measured by ELISA in a clinical laboratory.

### Cell culture

Hep-G2 human hepatocarcinoma cells (ATCC, Manassas, VA, USA) were cultured at 37 °C – 5% CO_2_ in Eagle’s Minimum Essential Medium (EMEM) (Wisent) supplemented with 10% fetal bovine serum (FBS) (Wisent), non-essential amino-acids (Wisent) and penicillin/streptomycin (Wisent).

### Inducible mouse model of SLE

Recommendations of the Canadian Council on Animal Care were followed in a protocol approved by the Animal Welfare Committee at Université Laval. C57BL/6 J were obtained from Jackson Laboratories (Bar Harbor, ME, USA) and housed in an Elite-Plus specific-pathogen-free animal facility at CHU de Quebec. SLE autoantibodies were induced in these mice as previously described^63^. In brief, 6–8-week-old male mice received 100 µL i.v. injections of β_2_GPI (20 µg) (Crystal Chem, Downers Grove, IL, USA) followed 24 h later by a 100 µL i.v. injection of lipopolysaccharide (LPS from *E. coli*, O111:B4; 10 µg) (Sigma-Aldrich, Saint-Louis, MO, USA). These injections were repeated after 2 and 4 weeks for a total of three rounds of immunizations and mice were bled 48 h after the third immunization. C57BL/6 J mice injected i.v. with PBS under the same schedule were used as controls.

### Ethics

Throughout the entire study, blood samples were obtained from patients under informed consent. All the methods presented in this study were performed in accordance with the relevant guidelines and regulations for both human and murine donors.

### Mitochondria isolation

Mitochondria were isolated from the livers of C57BL/6 J mice following a combination of published protocols^61,62^. Mice were sacrificed by cervical dislocation, the liver was swiftly removed, the gallbladder excised, and the liver was rinsed in ice-cold PBS (137 mM NaCl, 3 mM KCl, 19 mM Na_2_HPO_4_, 2 mM KH_2_PO_4_). The liver was then minced and transferred to a pre-cooled glass/Teflon tissue grinder containing 12 mL of ice-cold mitochondrial isolation buffer (10 mM Tris, 1 mM EGTA, 200 mM sucrose) per gram of liver then ground until an homogeneous suspension was obtained. Intact cells and nuclei were pelleted twice at 700 *g*, 4 °C, 10 min. Contaminating membranes, proteins and organelles were eliminated by two centrifugations at 7,000 *g*, 4 °C, 10 min followed by a centrifugation at 10,000 *g*, 4 °C, 10 min. The crude mitochondrial suspension was further purified by ultracentrifugation against a Percoll gradient (10 mM Tris, 1 mM EGTA, 30% v:v Percoll) at 95,000 *g*, 4 °C, 30 min. The band containing mitochondria was collected in PBS. A similar approach was used to isolate human mitochondria with the exception that up to 10^7^ Hep-G2 cells were lysed by repeated passages through a narrow-gauge needle. The commercial kit QIAgen Qproteome (QIAgen, Hilden, Germany) was also used, following the manufacturer’s instructions, to isolate mitochondria from Hep-G2 cells for quality comparison by western blotting with the aforementioned protocol. Isolated mitochondria were dosed by the bicinchoninic acid (BCA) method using BCA Protein Assay Kit (Thermo Fisher scientific, Waltham, MA, USA). Freshly isolated mitochondria were used in all experiments with the exception of the submitochondrial particles preparations for which pelleted mitochondria were kept at −80 °C until needed.

### Submitochondrial particles (SMP) preparation

SMP were prepared as described elsewhere^60^. Briefly, frozen mitochondria were thawed at room temperature and diluted to 10 mg of mitochondrial proteins in 10 mM 4-Morpholinepropanesulfonic acid (MOPS). Samples were then sonicated using a Fisher Sonic Dismembrator Model 500 (Thermo Scientific) at 20% maximal output for 20 seconds then kept on a salt-ice water bath for 10 minutes. This cycle was repeated nine times. Samples were then centrifuged at 16,000 *g*, 4 °C, 10 minutes in order to discard unbroken mitochondria and other unwanted debris. Supernatants were collected in a fresh tube and their volumes adjusted to 2 mL in 10 mM MOPS. Samples were then ultracentrifuged at 150,000 *g*, 4 °C for 45 minutes. Pelleted SMP were resuspended in SMP buffer (225 mM mannitol, 75 mM sucrose, 10 mM HEPES, 0.1 mM EDTA, pH 7.4) and dosed. SMP preparations were kept at −80 °C until needed.

### Red blood cells microparticles (_RBC_MP) preparation

Red blood cells (RBC) were isolated from the blood of healthy human volunteers by centrifugation at 282 *g* for 10 minutes at RT. Platelet-rich plasma and buffy coat were discarded, and the RBC fraction kept. RBC were counted (Cellometer Auto M10, Nexcelom Bioscience, Lawrence, MA, USA) and adjusted to a concentration of 5 × 10^8^ cells/mL in Tyrode’s buffer (12 mM NaHCO_3_, 127 mM NaCl, 5 mM KCl, 0.5 mM NaH_2_PO_4_, 1 mM MgCl_2_, 5 mM Glucose, 10 mM HEPES, pH 7.4). 10^9^ cells were then diluted in 45 mL double-distilled water and incubated for 5 minutes. The tonicity of the buffer was then balanced by addition of 5 ml of PBS 10X (filtered through a 0.22-μm membrane). Remnant RBC were removed by centrifugation at 1,300 *g* for 5 minutes at RT and supernatant was centrifugated at 18,000 *g* for 90 minutes at 18 °C. RBC microparticle pellet was then suspended in 500 μl PBS. Protein concentration was measured with BCA assay.

### Western blotting

After quantification, 25 µg of sample per lane were loaded onto a 12% polyacrylamide gel and underwent migration for 1 h 30 min at 100 V (constant voltage). Gels were then transferred overnight onto polyvinylidene difluoride membranes (PVDF. BioRad, Hercules, CA, USA) at 4 °C and 60 mA (constant current). Non-specific binding sites were blocked in Tris-buffered saline (TBS)−0.1% Tween20 containing 5% non-fat dry milk for 4 h at room temperature. Proteins of interest were labeled overnight at 4 °C with either mouse anti-actin (Clone AC-15, 1:5,000. Sigma-Aldrich), mouse anti-tubulin (Clone DM1A, 0.2 µg/mL. Abcam, Cambridge, UK), mouse anti-proliferating cell nuclear antigen (PCNA. Clone PC10, 1 µg/mL. Santa Cruz biotechnology, Santa Cruz, CA, USA), rabbit anti-VDAC (Clone D73D12, 1:1,000. Cell Signaling Technology, Danvers, MA, USA), mouse anti-TOMM22 (Clone IC9-2, 2 µg/mL. Abcam) or mouse anti-cytochrome C antibody (Clone 7H8.2C12, 0.5 µg/mL. BD Biosciences, Franklin Lakes, NJ, USA), polyclonal rabbit anti-proteasome 20 S (0.8 µg/mL. Abcam), polyclonal rabbit anti-GRP94 (1:2,000, Abcam) diluted in superblock (BioRad). Following a 1-h incubation with horseradish peroxidase-conjugated anti-mouse or anti-rabbit antibody (1:10,000 in superblock) (Jackson Immunoresearch, West Grove, USA), signals were visualized with Western Lightning Chemiluminescence Reagent (Perkin Elmer, Waltham, MA, USA) on a C-DiGit membrane scanner (LI-COR biotechnology, Lincoln, NE, USA). Total proteins isolated from starting materials (*i.e*. mouse liver or Hep-G2 cells) were used as controls. Full-length blots are presented in Supplementary Fig. 6a–c.

### Mitochondrial size measurement by dynamic light scattering (DLS)

The size of the isolated mitochondria was measured by DLS, using a Zetasizer Nano ZS device (Malvern instruments, Malvern, UK) equipped with the standard 4 mW, 633 nm He-Ne laser as a light source, set at a detection angle of 173°. Experiments were replicated three times in 1 cm length disposable UV-Cuvettes (Brand GMBH, Wertheim, Germany) containing 100 µL of isolated murine mitochondria diluted in PBS at a concentration of 10 µg proteins/µL. The following parameters were taken into account upon measuring the size of the mitochondrial: refraction index of the solvent: 1.330, viscosity of the sample: 0.8872 mPas, refraction index of the proteins: 1.45, temperature: 25 °C.

### Electron microscopy

Mitochondria (10^8^) were fixed in 3.5% acrolein for 15 min at room temperature. Fixed samples were rinsed twice in PBS then embedded in 4% agarose. 50 µm sections were cut using a vibratome, post-fixed in 1% osmium tetroxide for 30 minutes and embedded in Durcupan resin. Seventy-nanometer ultrathin sections were visualized at 80 kV using a Tecnai G2 Spirit BioTWIN (FEI, Hillsboro, OR, USA) transmission electron microscope.

### Assessment of the mitochondrial oxygen consumption

Mitochondria were resuspended in mitochondrial assay solution (MAS: 70 mM sucrose, 220 mM mannitol, 10 mM KH_2_PO_4_, 5 mM MgCl_2_, 2 mM HEPES, 1 mM EGTA and 0.2%(w/v) fatty acid-free BSA, pH 7.4 at 37 °C) and supplemented with 10 mM pyruvate, 2 mM malate and 4 μM FCCP [carbonyl cyanide 4-(trifluoromethoxy)phenylhydrazone], pH 7.4. An equivalent of 10 μg proteins was seeded on XF-96 plates (Agilent, Santa Clara, CA, USA). Plates were then centrifuged 2,000 *g* for 20 min at 4 °C. We visualized the distribution of mitochondria under a bright-field microscope to ensure adherence and homogeneous repartition. Plates were maintained at 37 °C without CO_2_ for approximately 40 minutes prior to loading. Oxygen consumption rates (OCR) were measured in accordance with manufacturer instructions (Agilent/Seahorse Bioscience). Experiments were replicated in three wells and averaged for each experimental condition. A total of 3 measurements of oxygen consumption for each condition were made approximately every 6 minutes under basal conditions and after sequential addition of rotenone (2 μM), succinate (10 mM), antimycin A (40 μM) and TMPD (*N,N,N*′,*N*′-tetramethyl-*p*-phenylenediamine)/Ascorbate (100 μM/10 mM). Succinate was used as the electron donor for complex II, rotenone as a complex I inhibitor, antimycin A as a complex III inhibitor and respiration through complex IV was measured using TMPD/ascorbate.

### High sensitivity flow cytometry

Due to their small size, mitochondria were detected by high sensitivity flow cytometry, using a “small particle option” consisting of a forward scatter (FSC) coupled to a photomultiplier tube (PMT) mounted on a BD FACS Canto II Special Order Research Product (SORP, BD Biosciences). Mitochondria (0.5 µg) were stained with 1 µg of anti-TOMM22-Atto 488 (clone IC9-2, Sigma-Aldrich) and 1 µM of mitotracker deep red (Invitrogen, Carlsbad, CA, USA) for 30 minutes at 37 °C and diluted with PBS to a final volume of 500 µl before flow cytometry analysis. To quantitatively measure the number of mitochondria, we used 3 µm diameter polystyrene microsphere (Polysciences, PA, USA). 80,000 microspheres were added to each sample and 500 microspheres were acquired. Silica particles (Kisker Biotech, Steinfurt, Germany) were used to determine 100–1,000 nm size scale.

### Mitochondrial DNA extraction by alkaline lysis

Isolated mitochondria were pelleted at 10,000 *g*, 4 °C, 10 min and resuspended in 0.8 mL mtDNA isolation buffer A [50 mM Tris-HCl, 10 mM EDTA, 100 µg/mL RNAse A (QIAgen), pH 7.5] for every 3 mg of mitochondrial proteins. Mitochondria were then lyzed in one volume (v:v) mtDNA isolation buffer B (extemporaneously-prepared. 0.2 M NaOH, 1% SDS) for 3 min. on ice, under gentle agitation. mtDNA suspensions were neutralized with one volume (v:v) mtDNA isolation buffer C (1.32 M potassium acetate, pH 4.8 adjusted with ice-cold acetic acid) for 5 min. on ice, under gentle agitation. Mitochondrial debris were pelleted by centrifugation for 10 minutes at 14,000 *g*, RT. Supernatants were transferred to fresh tubes. mtDNA was precipitated overnight at −20 °C with 0.1 volume (v:v) potassium acetate (stock solution: 3 M sodium acetate, pH adjusted to 5.2 with ice-cold acetic acid) and 0.7 volume (v:v) absolute isopropanol. mtDNA was then pelleted at 14,000 *g*, washed thrice with 1.5 mL 70% ethanol and resuspended in DNA resuspension buffer (10 mM Tris-HCl, 10 mM EDTA, pH 8.0). Mitochondrial DNA concentration was determined by spectrophotometry (Nanodrop 1000, Thermo Fisher Scientific).

### Nuclei isolation from mouse hepatocytes

Nuclei were isolated from mouse livers using published methods^86^. Briefly, during mitochondria isolation protocols, while supernatants were used for performing mitochondria isolations, pellets from the first 700 *g* centrifugation were resuspended in 10 mL of ice-cold nuclei isolation buffer A (5 mM MgCl_2_, 10 mM Tris–HCl, 250 mM Sucrose. pH 7.4) and ground in a pre-cooled glass/Teflon tissue grinder. The suspension was further disrupted by three passages through a 25 G^5^/_8_ gauge needle followed by two passages through a 27 G ½ gauge needle. The suspension was then filtered against a 40 µM nylon cell strainer (Thermo Fisher Scientific) and centrifuged at 600 *g*, 4 °C, 10 min. Pellets were resuspended in 14 mL buffer A and centrifuged at 600 *g*, 4 °C, 10 min. Pellets were then resuspended in 9 volumes of ice-cold nuclei isolation buffer B (1 mM MgCl_2_, 10 mM Tris– HCl, 2.0 M sucrose. pH 7.4) and centrifuged at 16,000 *g*, 4 °C, 30 min. Pellets were resuspended in 200 µL PBS and stored at −20 °C.

### Whole-cell and nuclear DNA isolation

DNA from either 25 mg of whole mouse liver or the 200 µL or isolated nuclei were extracted using QIAmp® DNA Mini Kit (QIAgen), following instructions from the manufacturer. Samples were eluted in DNA resuspension buffer.

### Mitochondrial DNA and nuclear DNA enrichments by qPCR

Thirty nanograms of mitochondrial DNA, nuclear DNA or the same amount of total DNA extracted from whole mouse liver using the aforementioned protocols, were amplified in a Rotor-Gene Q real time qPCR cycler (QIAgen) according to standard protocols with SsoAdvanced Universal SYBR^®^ Green Supermix (BioRad) in a 10 µL reaction volume. Two distinct primer couples were used: one specific to mitochondrial DNA (5′-GGAACAACCCTAGTCGAATGAA-3′/5′-GCTAGGGCCGCGATAATAAA-3′) and the other to nuclear DNA (5′-CCTGCTGCTTATCGTGGCTG-3′/5′-GCCAGGAGAATGAGGTGGTC-3′). The experimental conditions were: 50 °C - 2 minutes in, 95 °C - 10 min and 40 cycles (95 °C - 15 seconds, 60 °C - 1 minutes). Fold changes were calculated with the 2^−ΔCt^ method setting mean value for total liver DNA extracts as 1.

### Mitochondrial DNA digestion by restriction enzymes

Purified mouse mtDNA was digested by *Hae II* or *Pst I* restriction endonucleases (NEB, Whitby, ON, Canada) according to the manufacturer’s protocol. Digested products (1.5 μg) were then resolved on a 0.5% (w/v) agarose gel. Images were acquired on a Chemidoc MP gel documentation system (Bio-Rad). Full-length gel is presented in Supplementary Fig. 6d.

### S1 nuclease treatment of mitochondrial DNA and nuclear DNA

30 µg of mtDNA and nDNA were diluted in 200 µL of 1X S1-buffer and treated with 50 U S1 nuclease (Thermo Fischer Scientific) for 5 minutes at 37 °C. Reaction was stopped by addition of 600 mM EDTA (final concentration) and incubation at 70 °C for 10 minutes. Samples were precipitated by addition of 300 mM Sodium Acetate (final concentration) and 2 volumes (v:v) absolute ethanol for 16 hours at −20 °C. Samples were pelleted at 14,000 *g*, RT, 20 minutes, and washed 1 mL 70% ethanol and resuspended in DNA resuspension buffer. For competition assays, untreated nDNA and mtDNA followed the same treatment without addition of the enzyme. Samples were dosed by qPCR.

### Mitochondria oxidation *in-vitro*

Dosed mitochondria were pelleted and resuspended at a concentration of 1.5 mg of mitochondrial proteins/mL in PBS containing 500 µM of the oxidant tertbutyl hydroperoxide (TBHP) (Sigma-Aldrich). Mitochondria were oxidized for 1 h 30 min at 37 °C under gentle agitation then rinsed twice with ice-cold PBS. Oxidized mitochondria were subsequently quantified by BCA.

### Mitochondrial lipid oxidation

Thiobarbituric acid reactive substances (TBARS) formation was assessed, using the TBARS Parameter Kit (R&D systems, Minneapolis, MN, USA), following the manufacturer’s instructions. Two hundred micrograms of mitochondria were lysed, the proteins were precipitated using trichloroacetic acid (TCA) and pelleted at 12,000 *g*, room temperature, 4 min. The supernatants were transferred to fresh tubes. Samples (75 µL) were incubated with 37.5 µL of thiobarbituric acid in a 96-well plate for 3 h at 50 °C. Absorbances were read at 532 nm on a SpectraMax 190 microplate reader (Molecular Devices, Sunnyvale, CA, USA) and TBARS quantification was performed using a standard curve provided in the kit. Oxidized samples were compared to control mitochondria that were incubated under the same conditions in PBS devoid of TBHP.

### Mitochondrial protein oxidation

The formation of carbonyl moieties following *in-vitro* mitochondrial oxidation was measured using the Protein Carbonyl Content Assay Kit (Sigma Aldrich) following the manufacturer’s instructions. Oxidized samples were compared to control mitochondria that were incubated under the same conditions in PBS devoid of TBHP.

### Detection of antibodies targeting mitochondrial epitopes by ELISA

For the detection of anti-whole mitochondrial antibodies (AwMA), murine mitochondria were diluted (500 µg/mL) in 50 mM carbonate/bicarbonate buffer, pH 9.6 and 25 µL per well were loaded onto 96-well half-area clear flat bottom polystyrene high-binding microplates (Corning, New York, USA). Plates were coated for 18 h at 4 °C then blocked for 4 h at 37 °C with PBS containing 10% FBS and 0.5% gelatin. After three washes with PBS, sera diluted 1:150 (unless otherwise specified) in PBS-10% FBS-0.3% gelatin were incubated overnight at 4 °C in duplicate. After three washes with PBS, plates were incubated for 1 h at room temperature with alkaline phosphatase-(AP) conjugated goat anti-mouse or anti-human IgG (Sigma-Aldrich) diluted 1:1,000 in PBS-0.4% bovine serum albumin (BSA). Plates were washed thrice with PBS and developed with *p*-nitrophenol phosphate (*p*-NPP) for ~30 min at 37 °C and optical densities (OD) were read at 405 nm on a microplate reader. The same protocol was used for the detection of autoantibodies targeting submitochondrial particles by using 25 µL per well of SMP diluted (50 µg/mL) in 50 mM carbonate/bicarbonate buffer, pH 9.6. A similar approach was used for human mitochondria with the following modification: plates were incubated with a horseradish peroxidase-conjugated anti-human IgG secondary antibody (1:3,000), peroxidase activity was revealed at room temperature for ~5 min with 3,3′,5,5′-tetramethylbenzidine (TMB). The reaction was stopped with 2 N H_2_SO_4_ and the ODs read at 450 nm. For the anti-mtDNA ELISA, plates were pre-coated with 1% protamine sulfate in double-distilled water for 1 h at room temperature. Plates were washed three times with PBS and coated overnight at 4 °C with 400 ng mtDNA in PBS. All subsequent steps were identical to those used for AwMA-ELISAs. Blank values (mitochondrial antigens and no sera) were substracted from measured values for each patient. During the development of these assays, the proper coating of the wells was tested by using isotype-matched mouse monoclonal antibodies (mAb). A monoclonal antibody (Clone IV.3, 4 µg/mL) was used as a negative assay control in each instance while different monoclonal antibodies were used, depending on the coating antigens, as positive assay controls: an anti-TOMM22 mAb (Clone IC9-2, 4 µg/mL. Abcam) for intact mitochondria, an anti-DNA mAb (Clone 35I9 DNA, 10 µg/mL. Abcam) or an anti-Cytochrome C mAb (Clone 7H8.2C12, 5 µg/mL. BD Biosciences).

### Competition assay

AwMA-ELISAs were performed as described in the previous sections with the following modifications: serum samples were pre-incubated in dilution buffer (1:150) spiked with various concentrations (0.25, 1 and 3 mg/mL) of competitors (i.e. mitochondria or red blood cells microparticles) for 3 hrs. at RT and incubated in duplicate overnight at 4 °C. Data are presented as the percentage of signal remaining after each competition, compared to the OD (405 nm) measured in absence of competitors.

A similar procedure was used for AmtDNA-ELISA, using increased concentrations (0, 3, 9 and 27 ng/µL) of competing DNA (extracted from nuclei or mitochondria, with or without S1 nuclease treatment).

### Statistics

Comparisons between groups were made using either Student’s t-test, Wilcoxon test, Friedman or Kruskal-Wallis tests, one-way ANOVA, two-way or repeated measures ANOVA depending on the outcome, as well as the number and type of groups. When multiple comparisons were assessed, appropriate post-hoc correction tests were used such as Dunn’s, Dunnett’s, Sidak’s or Bonferroni’s. Associations between AwMA, AmtDNA and anti-HSP60 were computed with Spearman correlations. Distribution of these antibodies according to ACA results were compared using Wilcoxon test. Associations between AwMA or AmtDNA and clinical outcomes were studied by bivariate and multivariate linear and logistic regressions. Clinical outcomes studied are average intima media thickness, percent change of the flow mediated dilatation (FMD) of the brachial artery, presence of FMD, presence of plaque in the carotid, thrombotic event ever, white blood cells count, platelet count, increased DNA binding by Farr assay above normal range for testing laboratory, presence of damage according to SLICC Damage Index (SDI > 0), high activity according to SLEDAI-2K activity score (SLEDAI ≥ 4), presence of lupus nephritis, biopsy class, as well as chronicity and activity index from the biopsy. The latter were adjusted for disease duration, age, body mass index (BMI), low-density lipoproteins (LDL) cholesterol, antimalarial medication and prednisone. Logistic regressions are presented with odd ratios and their 95% Wald confidence interval. Participants’ results were considered positive for AwMA and AmtDNA when their value was above the cut-off value identified after maximizing Youden’s Index. A 95% confidence interval was obtained for the cut-off using 10,000 bootstrap samples. Performance measures are presented with their 95% exact confidence interval.

### Software

Western blot images were acquired using Image Studio Digits 5.2 (LI-COR biotechnology). mtDNA migration through agarose gel was imaged using Image Lab 6.0.1 (Bio-Rad). DLS studies were carried using the built-in Zetasizer 7.10 software (Malvern Instruments). EM images were acquired with the Image Capture Software 601.384 (Advanced Microscopy Techniques Corp., Woburn, MA, USA). Flow cytometry was performed using the BD FACSDiva™ 6.1.3 (BD Biosciences). Yields from DNA isolations were quantified using the ND-1000 3.8.1 software (Thermo Fisher Scientific) and qPCR were performed with RotorGene 6.1 (Corbett Research/QIAgen). Optical densities were measured using SoftMax Pro 5.4.1 (Molecular Devices). Figures were assembled with ImageJ 1.47 (National Institutes of Health, Rockville, MA, USA) and Photoshop CS6 13.0 (Adobe Systems Inc., Mountain View, CA, USA). Statistical analyses were carried with Prism 7 software (GraphPad Software Inc., La Jolla, CA, USA) and SAS version 9.4 (SAS Institute Inc., Cary, NC, USA).

## Supplementary information

Supplementary material

## Data Availability

The datasets generated during and/or analyzed during the current study are available from the corresponding author on reasonable request.
