# Supplementary material for: Anti-mitochondrial autoantibodies in systemic lupus erythematosus and their association with disease manifestations
Source: Sci Rep. 2019 Mar 14;9:4530. doi: 10.1038/s41598-019-40900-3 (PMC6418244; doi:10.1038/s41598-019-40900-3)
Supplement: Supplementary file 1 — Supplementary material [file 41598_2019_40900_MOESM1_ESM.docx]

**Supplementary information:**

**Anti-mitochondrial autoantibodies in systemic lupus erythematosus and their association with disease manifestations**

Yann Becker, Renée-Claude Loignon, Anne-Sophie Julien, Geneviève Marcoux, Isabelle Allaeys, Tania Lévesque, Emmanuelle Rollet-Labelle, Hadrien Benk-Fortin, Nathalie Cloutier, Imène Melki, Lihi Eder, Éric Wagner, Martin Pelletier, Hassan El Hajj, Marie-Ève Tremblay, Clémence Belleannée, Marie-Josée Hébert, Mélanie Dieudé, Joyce Rauch, Paul R. Fortin, and Éric Boilard

**Supplementary figures:**

**
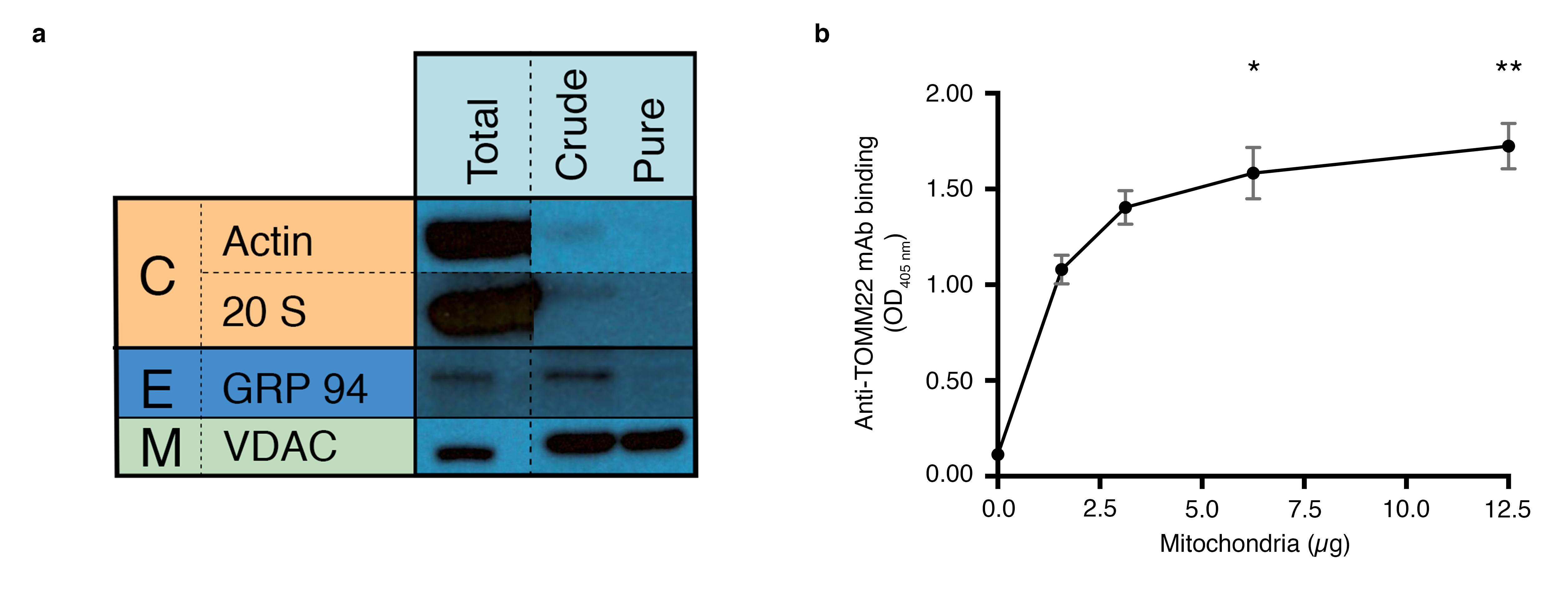
**

**Supplementary Figure 1. Isolation of highly purified isolated mitochondria**

(a) Crude mitochondria carry contaminants from the endoplasmic reticulum (E) and from the proteasome (20S) that are lost upon ultracentrifugation using a Percoll gradient, yielding more highly purified mitochondria (pure mitochondria). Results are representative of three distinct preparations. Blots (20 µg protein per lane) separated by the dashed line are non-contiguous but from same membrane. (b) Wells were coated in quadruplicate with increasing amounts of isolated mitochondria, ranging from 1.6 to 12.5 µg, and incubated with a murine anti-TOMM22 monoclonal antibody (clone IC9-2, 4 µg/mL). Antibody binding was detected with an alkaline-phosphatase conjugated goat anti-mouse IgG and revealed with p-nitrophenol phosphate for 30 minutes. ODs were read at 405 nm. Friedman test with multiple comparisons (Dunn’s correction) OD detected were compared to the OD measured in wells without mitochondria. All experiment presented in the figure were performed using mouse mitochondria. Data are mean ± SD. *p<0.05. **p<0.01. C: cytosolic markers; Crude: crude mitochondria; GRP 94: 94 kDa glucose-related protein; M: mitochondrial markers; mAb: monoclonal antibody; Pure: pure mitochondria; Total: Total liver; VDAC: voltage-dependent anion channel; 20S: 20S proteasome.

**
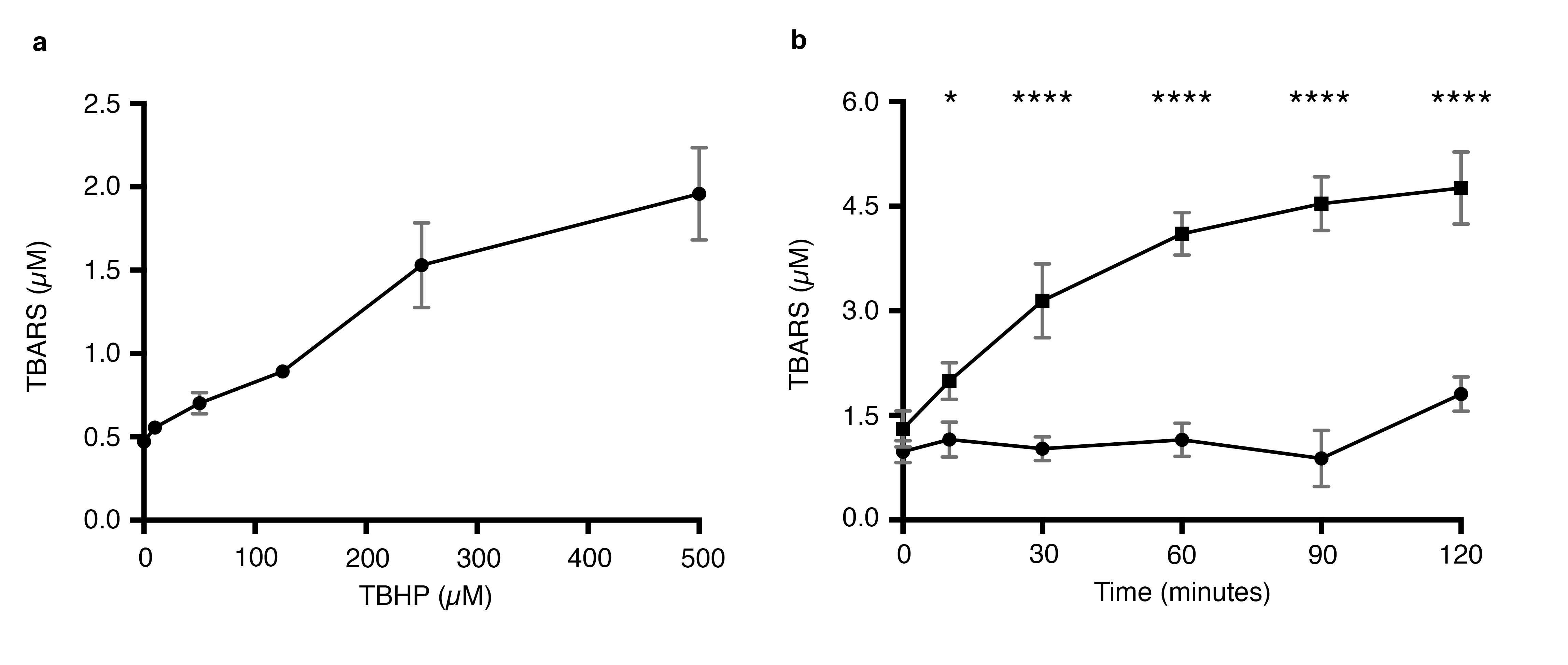
**

**Supplementary Figure 2. Assessment of mitochondrial oxidation.**

(a) Isolated mitochondria (n = 3) were treated with increasing concentrations of tert-buthyl hydroperoxide (TBHP) (10 - 500 µM in PBS) and lipid oxidation was quantified using a commercial kit. Friedman test with multiple comparisons (Dunn’s correction). (b) The optimal duration of the oxidative treatment was determined by the kinetics of the lipid oxidation (10 - 120 min) with 500 µM TBHP (filled squares), as detected with a commercial kit (n = 3), and compared with mitochondria resuspended in PBS buffer devoid of TBHP (filled circles). Statistical differences were determined by two-way repeated measures ANOVA with multiple comparisons (Bonferroni’s correction) between native and oxidized mitochondria for each time point. All experiment presented in the figure were performed using mouse mitochondria. Data are mean ± SD. Data shown are the mean ± SD of three samples. *p<0.05. ****p<0.0001.


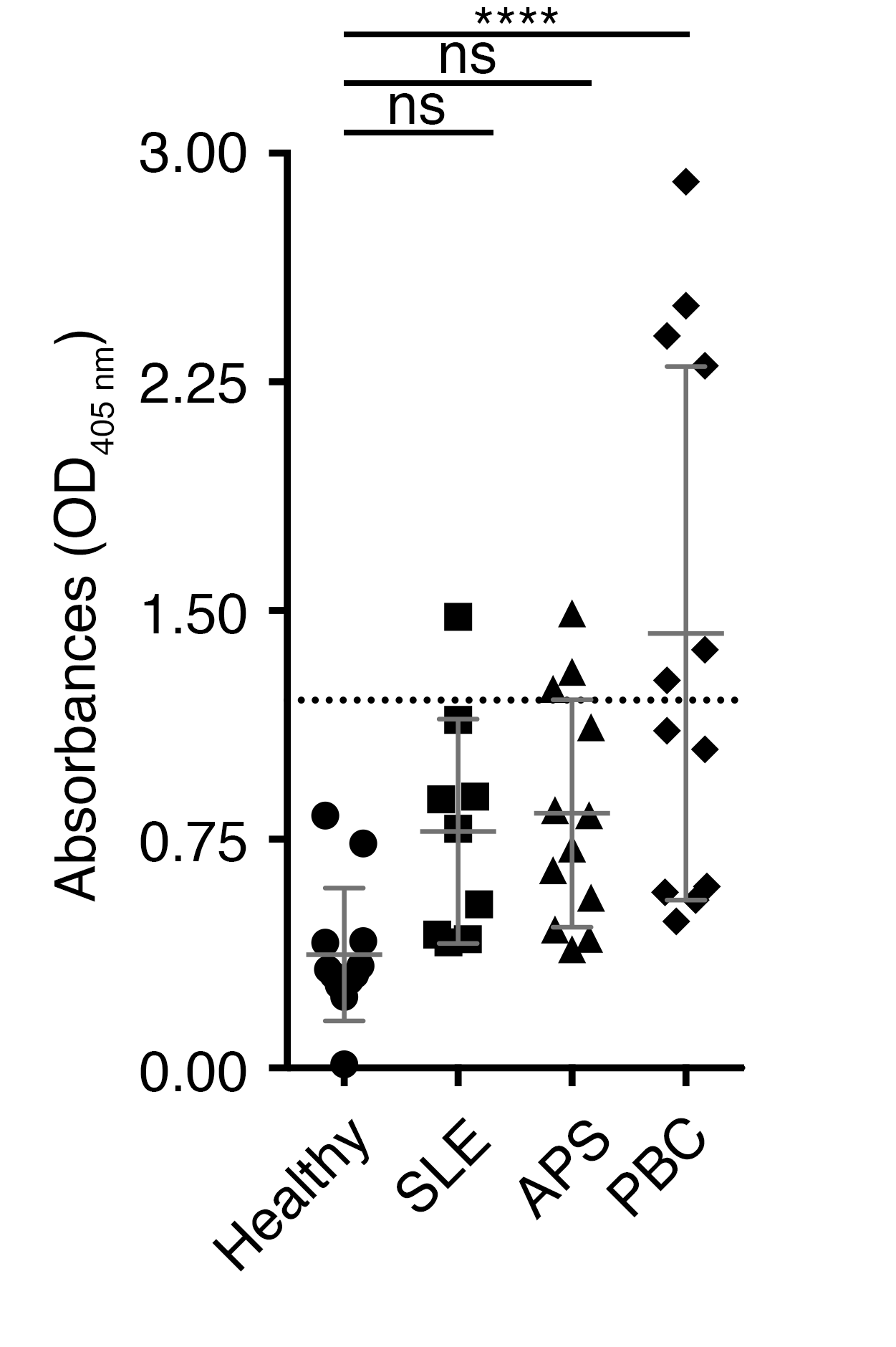


**Supplementary Figure 3. Detection of autoantibodies targeting submitochondrial particles.**

Direct ELISA was performed using murine submitochondrial particles (1.25 µg) as coating antigen. A significant elevation in the autoantibody titer was detected in sera (1:150) from primary biliary cirrhosis (PBC) patients when compared to sera from healthy donors, but not in sera from patients suffering from systemic lupus erythematosus (SLE) or antiphospholipid syndrome (APS). An isotype-matched monoclonal mouse anti-cytochrome C antibody (clone 7H8.2C12, 5 µg/mL) was included as a positive assay control (dotted line). Healthy donors: n = 12; SLE: n = 9; APS: n = 12; PBC: n = 12. Statistical differences were determined by one-way ANOVA test with multiple comparisons (Dunnett’s correction) to healthy donors. Data shown are the mean ± SD for each group. Not significant (ns): p>0.05. ****p<0.0001.

^
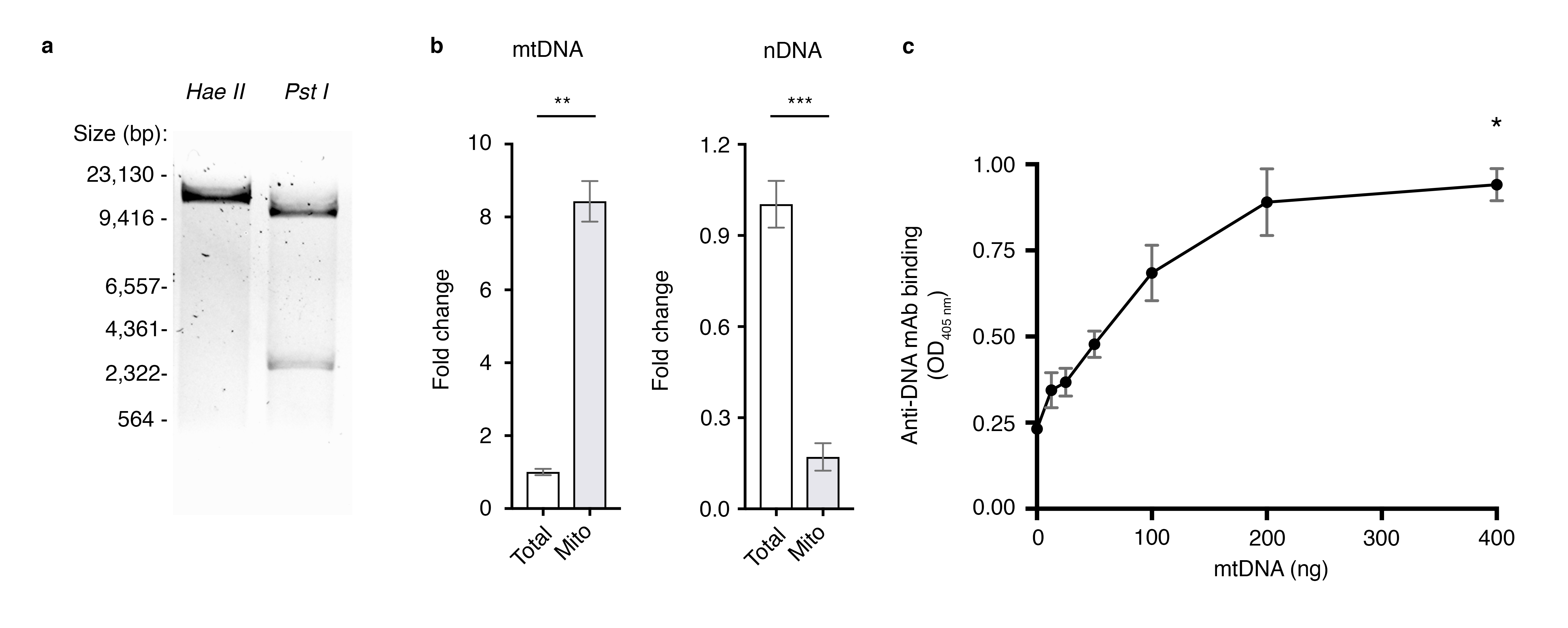
^

**Supplementary Figure 4. Assessment of the isolated mitochondrial DNA purity**

(a) 1.5 μg of mtDNA was digested by two different restriction enzymes; *Hae II* (left lane) or *Pst I* (right lane). Fragments were resolved on a 0.5 % agarose gel. (b) Mitochondrial DNA (mtDNA) (left panel) and nuclear DNA (nDNA, right panel) were amplified by qPCR to assess the presence of contaminating nucleic acids originating from the nucleus. mtDNA isolated from isolated mitochondria (Mito) was compared to total DNA extracted from mouse liver (Total). N = 3, Data were analyzed by One sample Student’s t-tests. (c) Saturation of protamine-sulfate pre-coated wells by mtDNA was performed using increasing doses of mtDNA (12.5 - 400 ng per well in triplicate) incubated with a mouse anti-DNA monoclonal antibody (Anti-DNA mAb, clone 35I9 DNA, 10 µg/mL). Friedman test with multiple comparisons (Dunn’s correction) to the OD values measured in wells with no mtDNA. All experiment presented in the figure were performed on murine samples. Data are mean ± SD. *p<0.05. ** p<0.01. *** p<0.001. Mito: mtDNA isolated from mitochondrial samples; total: DNA extracted from total mouse liver.

**
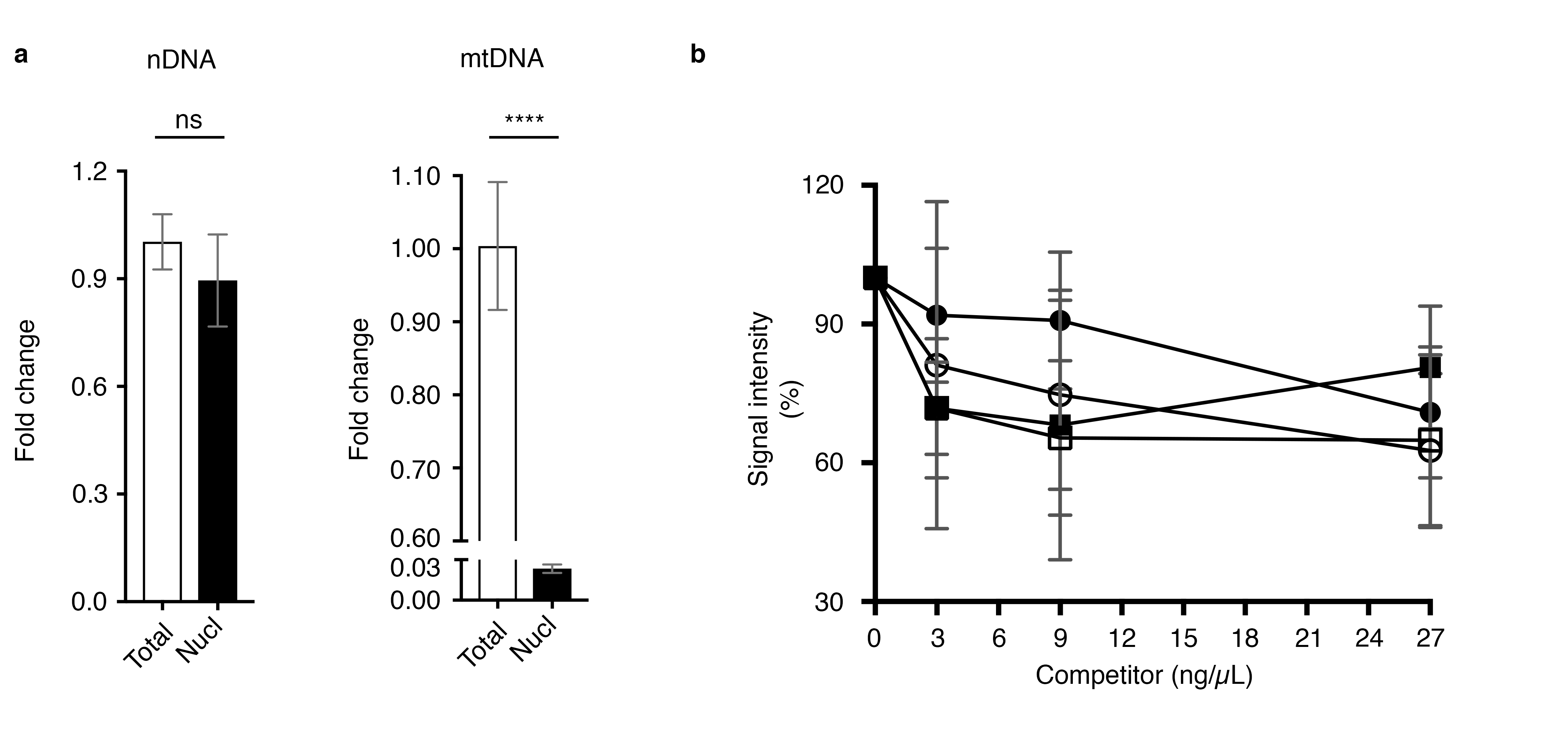
**

**Supplementary Figure 5. AmtDNA display cross-reactivities between mitochondrial and nuclear DNA.**

(a) Quality of the nDNA isolated from mouse hepatocytes was assessed by qPCR using primers specific to nuclear DNA (nDNA, left panel) or to mitochondrial DNA (mtDNA, right panel). DNA samples isolated from nuclei (Nucl) were compared to total DNA extracted from mouse liver (Total). N = 3, Data were analyzed by One sample Student’s t-tests. (b) Cross-reactivities of AmtDNA was assessed by competitive ELISA (N=9 SLE patients per condition). Increased concentrations (0, 3, 9 and 27 ng/µL) of mtDNA (circles) or nDNA (squares), treated (filled symbols) or not (hollow symbols) with S1 nuclease were used as competitors. Increased concentrations of competitors significantly decreased AmtDNA binding to the assay (****, p<0.0001). However, no significant differences were found between the competitors’ effects. Two-ways ANOVA with repeated measures. All experiment presented in the figure were performed on DNA isolated from mouse liver. Data are mean ± SD. Not significant (ns): p>0.05. **** p<0.0001. Nucl: nDNA purified from nuclei isolated from murine hepatocytes; total: DNA extracted from total mouse liver.


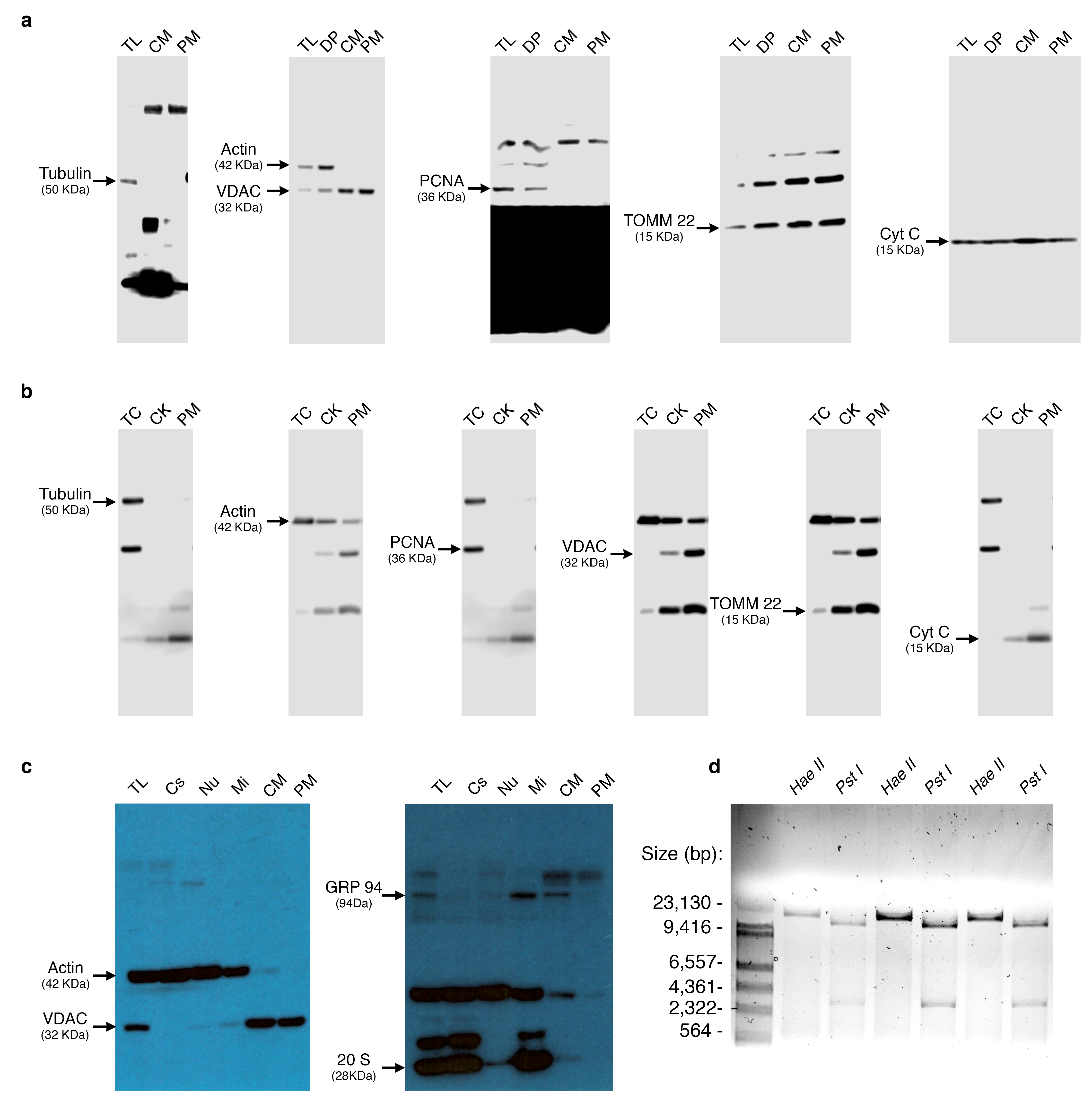


**Supplementary Figure 6. Full length images for western blots and agarose gels.**

Image corresponding to samples from various steps of the murine and human protocols for mitochondria isolation were acquired using the Image Studio Digit software on high sensitivity setup (maximal acquisition time: 12 min.), brightness and contrast were adjusted on the machine, as shown, for each protein of interest. Images were subsequently cropped and assembled as presented in **Figure 1 b** with no further editing. (a) The quality of the murine preparations was assessed by blotting (25 µg protein per lane) protein lysates obtained: from total liver (TL); the pellet obtained after the first 700 *g* centrifugation step containing unbroken cells and nuclei (DP); crude mitochondria (CM); and pure mitochondria (PM), results from TL and PM were presented in the figure. (b) Two different methods for the isolation of human mitochondria from Hep G2 cells were tested using total cell lysates (TC): one from a commercial kit (CK) and the other following the protocol described in the Material and Methods section (PM). Results from TC and PM are presented in the figure. The commercial kit was not used for any other results obtained in this study. (c) Differences between crude and pure mitochondria isolated from mouse liver were assessed. Images were acquired analogically on X-ray film, developed and scanned. Several cellular sub-compartments were tested [i.e. total liver (TL), cytosol (Cs), nuclei (Nu), microsomes (Mi), crude (CM) and pure mitochondria (PM)] but only TL, CM and PM are presented in the **supplementary figure 1 a**. (d) Mitochondrial DNA digestion by restriction enzymes, acquired using the Image Lab software and presented in the **supplementary figure 4 a**. From left to right: DNA ladder, *Hae II*-digested sample, *Pst I*-digested sample. PCNA: proliferating cell nuclear antigen; TOMM22: translocase of the outer mitochondrial membrane; VDAC: voltage-dependent anion channel.

**Supplementary tables:**

**Supplementary Table 1. Additional demographics and clinical variables for SLE patients included in the study (n=175)**

| **Demographic variables** | **SLE patients** |
| --- | --- |
| Ethnicity, n (%) (n=174)  Caucasian  Asian  Black  Others | 99 (57)  36 (21)  31 (18)  8 (5) |
| Body Mass Index (BMI)  Range  Mean ± S.D | 15-55  26 ± 6 |
| Average carotid intima-media thickness (n = 103)  Range, µm  Mean ± S.D, µm | 388-969  611 ± 104 |
| Percent change of the flow-mediated dilatation, brachial artery (n = 72)  Range, percent  Mean ± S.D, percent | -8.7-34.3  8.5 ± 8.8 |
| Flow mediated dilatation of the brachial artery, n (%) | 82 (47) |
| Presence of plaque in the carotid, n (%) (n = 103) | 36 (35) |
| Biopsy class (n=62), n (%)  0  1  2  3  4  5  6  45 | 1 (2)  4 (7)  11 (18)  13 (21)  18 (29)  12 (19)  1 (2)  2 (3) |
| Activity Index from the biopsy (n = 58)  Range  Mean ± S.D | 0-11  4 ± 3 |
| Chronicity Index from the biopsy (n = 58)  Range  Mean ± S.D | 0-9  2 ± 2 |

LDL: low density lipoproteins.

^A^: Reference range 150 - 400.

^B^: Reference range 4.0 - 5.1.

**Supplementary Table 2: Serologic variables for SLE patients**

| **Serologic parameters** | **SLE patients**  **(n = 175 except when specified)** |
| --- | --- |
| Cholesterol total (n=173)  Range, mmol/L  Mean ± S.D, mmol/L | 2.75-6.52  4.40 ± 0.81 |
| LDL cholesterol (n=169)  Range, mmol/L  Mean ± S.D, mmol/L | 0.94-3.99  2.37 ± 0.60 |
| HDL cholesterol (n=169)  Range, mmol/L  Mean ± S.D, mmol/L | 0.78-2.90  1.54 ± 0.42 |
| TC/HDL cholesterol (n=169)  Range, mmol/L  Mean ± SD, mmol/L | 1.65-6.73  3.01 ± 0.79 |
| PlateletsA (n=170)  Range, x10^9^/L  Mean ± S.D, x10^9^/L | 17-573  242.57 ± 74.53 |
| White blood cellsB (n=170)  Range, x10^9^/L  Mean ± S.D, x10^9^/L | 1.7-12.3  5.63 ± 2.10 |
| DNA Farr (n=174)  Range, U/mL  Mean ± SD, U/mL  < 7, n (%) | 1-101  15.90 ± 27.77  102 (58.6) |
| Increased anti-dsDNA antibodies, n (%) | 59 (33.7) |

^A^: Reference range 150 - 400.

^B^: Reference range 4.0 - 5.1.

TC: total cholesterol. DNA Farr: quantification of anti-dsDNA antibodies by Farr assay. HDL: high density lipoproteins. LDL: low density lipoproteins.

**Supplementary Table 3: Autoantibodies detected in SLE patients**

| **Variable of interest** | **SLE patients**  **(n=175 except when specified)** |
| --- | --- |
| Anti-HSP60 (n=172)  Range  Mean ± SD | 0.00-1.81  0.60 ± 0.38 |
| AmtDNA mean  Range  Mean ± SD | 0.08-2.45  0.45 ± 0.33 |
| AwMA mean  Range  Mean ± SD | 0.06-3.25  0.38 ± 0.30 |
| Lupus anticoagulant, n (%) (n=168) | 19 (11.3) |
| ACA positive, n (%) | 14 (8.0) |

ACA: anti-cardiolipin antibodies within 1 year of assessment. AwMA: anti-whole mitochondria antibodies. AmtDNA: anti-mitochondrial DNA antibodies, HSP60: 60 KDa heat-shock protein.
